# Utilization of Volume-Targeted Ventilation in Neonatal Intensive Care Units in the United Arab Emirates: A Survey of Practicing Neonatologists

**DOI:** 10.7759/cureus.111931

**Published:** 2026-07-01

**Authors:** Dhabya Al-Sultani, Ghada Kraism, Aimen Ben Ayad

**Affiliations:** 1 Neonatal Intensive Care Unit (NICU), Tawam Hospital, Abu Dhabi, ARE; 2 Neonatal Intensive Care Unit (NICU), Danat Al Emarat Women and Children’s Hospital, Abu Dhabi, ARE

**Keywords:** bronchopulmonary dysplasia, cross-sectional survey, high-frequency oscillatory ventilation (hfov), lung-protective ventilation, mechanical ventilation, neonatal intensive care, neonatology, tidal volume, united arab emirates (uae), volume-targeted ventilation

## Abstract

Background: Volume-targeted ventilation (VTV) is an evidence-based, lung-protective strategy demonstrated to reduce the incidence of bronchopulmonary dysplasia (BPD) and severe neurologic injury in premature infants. While variations in VTV adoption are well-documented in North America and Europe, data characterizing its utilization across the broader Middle East remains limited.

Objective: To evaluate the utilization rates of VTV, the selection of tidal volume (VT) targets across various clinical scenarios, and the perceived barriers to VTV implementation among practicing neonatologists in the United Arab Emirates (UAE).

Methodology: A cross-sectional survey was distributed to practicing neonatologists across multiple regions in the UAE. The survey assessed clinician and unit characteristics, core ventilation practices, scenario-based VT targeting, and barriers to VTV implementation.

Results: A total of 94 responses were analyzed. VTV was the most commonly reported invasive mode, with 78 (84.8%) of respondents classified as regular VTV users. VTV was predominantly utilized across both the acute and weaning phases of ventilation, with 60 (64.5%) respondents reporting its use. The reported median VT limits ranged from 4.0 to 6.0 mL/kg. Scenario-based responses revealed a median initial VT target of 5.0 mL/kg for a 500-g, 24-week infant with respiratory distress syndrome (RDS), 5.0 mL/kg for a term infant with meconium aspiration syndrome (MAS), and 4.0 mL/kg for an infant with congenital diaphragmatic hernia (CDH). High-frequency oscillatory ventilation (HFOV) was widely used (78, 97.5%); however, the use of volume guarantee (VG) during HFOV remained uncommon (28, 36.4%). Regular VTV use was significantly associated with ≥10 years of experience (*P* = 0.011) and practice in an academic/training level III NICU (*P* = 0.021). Unlike North American cohorts, the primary reported barrier to VTV adoption in the UAE was equipment unavailability rather than a lack of clinical understanding.

Conclusions: The UAE demonstrates a high rate of VTV adoption, aligning with the most progressive international benchmarks. However, scenario-specific VT targeting reveals a tendency to default to standard volumes regardless of the underlying lung pathophysiology. Continued efforts should focus on optimizing equipment availability and providing targeted education on individualizing VT settings to further elevate neonatal respiratory care.

## Introduction

Mechanical ventilation remains an essential and life-saving tool in the care of critically ill and extremely low birth weight (ELBW) infants, despite continuous improvements in non-invasive respiratory support [1]. However, prolonged or inappropriate use of invasive mechanical ventilation inherently exposes the immature lung to ventilator-induced lung injury (VILI) and the subsequent development of BPD [2]. Traditionally, the cornerstone of neonatal respiratory support has been time-cycled, pressure-limited ventilation (PLV) [3]. While PLV directly controls peak inspiratory pressure, it allows the delivered VT to fluctuate dramatically in response to rapid changes in pulmonary compliance and patient respiratory effort [4]. Compelling experimental and clinical evidence has shifted the understanding of VILI from barotrauma to volutrauma, revealing that excessive VTs and alveolar overdistension-rather than high airway pressures alone-are the primary determinants of lung injury [5].

To mitigate the risks of volutrauma, atelectrauma, and inadvertent hypocarbia, modern microprocessor-controlled ventilators now offer VTV modes, such as VG [4]. By continuously measuring exhaled VT and automatically adjusting the working inspiratory pressure to deliver a clinician-set target VT, VTV provides a dynamic, lung-protective strategy [3]. A robust and growing body of Level I evidence supports several clinically important advantages of VTV over traditional PLV. A comprehensive Cochrane systematic review by Klingenberg et al. concluded that VTV significantly reduces the combined outcome of death or BPD, as well as the incidence of pneumothorax, hypocarbia, severe intraventricular hemorrhage (IVH), and periventricular leukomalacia (PVL) [6]. Similarly, a meta-analysis by Peng et al. demonstrated that VTV significantly decreases the duration of mechanical ventilation and minimizes the failure rate of the primary assigned ventilatory mode [2]. The certainty of the evidence varies across outcomes and has generally been graded as low to moderate according to the GRADE (Grading of Recommendations Assessment, Development and Evaluation) framework, reflecting the limited number of events and methodological limitations of the contributing trials.

Despite clear physiological rationales and practical guidelines for bedside implementation [4], the global adoption of VTV into routine neonatal intensive care remains surprisingly inconsistent. An international survey by Klingenberg et al. revealed that while VTV was routinely used by half of the tertiary neonatal units in Australasia and the Nordic countries, European usage lagged significantly at approximately 11% [7]. More recently, Gupta and Keszler highlighted a stark contrast in North American practices, noting that 81% of Canadian neonatologists utilized VTV as their primary mode, compared to only 39% in the United States [1]. The most frequently reported barriers to VTV implementation include a lack of appropriately equipped ventilators and a profound lack of clinician understanding regarding appropriate VT targeting [1].

Expert guidelines stress that successful VTV requires a thorough understanding of the underlying lung pathophysiology and the individualization of ventilator settings, because *one size does not fit all* [8]. While VTV usage and barriers have been well-documented in North America, Europe, and Australasia, there is a critical lack of data characterizing the ventilation practices of neonatologists in the Middle East. The UAE features a rapidly advancing neonatal healthcare infrastructure with multiple level II and III NICUs, yet the extent to which VTV has been adopted into routine clinical practice remains unknown.

Therefore, this study aims to evaluate the utilization of VTV among practicing neonatologists across the UAE. By exploring core ventilation practices, the selection of VT targets across various clinical scenarios, and the perceived barriers to implementation, this survey seeks to bridge the knowledge gap regarding lung-protective ventilation strategies in the region.

## Materials and methods

Study design and participants

This cross-sectional questionnaire-based survey evaluated invasive ventilation practices among practicing neonatologists in the UAE, with emphasis on the use of VTV, VT target selection, and barriers to implementation. The survey was administered electronically through SurveyMonkey and distributed as a web link to neonatologists practicing across the UAE regions. Participants were identified and recruited through national and regional neonatal professional networks: the survey link was disseminated to practicing neonatologists via hospital and departmental NICU networks, neonatal professional-society mailing/email lists, and direct electronic invitations, including secure clinician WhatsApp groups. The total number of clinicians who received or opened the link was not recorded; therefore, a response rate could not be calculated. Responses were eligible if they were submitted by UAE-based practicing neonatologists and contained usable survey data. All 94 submitted responses were retained for analysis, with item-level denominators varying according to response completion. The survey questionnaire was adapted from previously published VTV practice surveys and modified for UAE practice.

Survey instrument

The questionnaire assessed conventional ventilators used for ELBW and very low birth weight (VLBW) infants, preferred invasive ventilation modes, frequency and timing of VTV use, monitoring and documentation of delivered VT during pressure-controlled ventilation, VT limits during VTV, scenario-based initial VT targets, synchronized ventilation modes during acute and weaning phases, perceived advantages and barriers to VTV, NICU characteristics, and high-frequency oscillatory ventilation (HFOV) practices, including use of VG or VT targeting during HFOV. The complete questionnaire, including all items and their response options, is provided in full as supplementary material in the Appendix.

Data preparation and definitions

The SurveyMonkey export used a two-row header structure, with parent questions in the first row and answer options or subquestions in the second row. The dataset was restructured into an analysis-ready format by combining parent-question and option labels into clear variable names while preserving the original responses. Select-all-that-apply items were analyzed as separate option-level indicators. Numeric VT responses were converted to milliliters per kilogram when interpretable; non-numeric or uninterpretable entries were treated as missing. No imputation was performed, and available-case denominators were used throughout.

Primary outcome and grouping variable

The primary outcome was the reported frequency of VTV use. For comparative analyses, respondents reporting VTV use most of the time (>60%) or often (30%-60%) were classified as regular VTV users; those reporting occasional use (20%-30%), rare use, or no use were classified as non-regular VTV users. This dichotomization followed the predefined ordinal response categories of the survey, with the 30% cut-point corresponding to the boundary between the *often* (30%-60%) and *occasionally* (20%-30%) options. Clinically, this threshold separates neonatologists who apply VTV as a routine, default approach in a substantial proportion of ventilated infants from those who reserve it for selective or infrequent use, and it avoids the unstable subgroups that would result from splitting the narrow intermediate categories. Practice location, years of neonatology practice, NICU bed capacity, and practice environment were grouped according to the response categories used in the survey.

Statistical analysis

Categorical variables were summarized as counts and percentages. Continuous VT variables were summarized as median (interquartile range) because their distributions were non-normal. Normality was assessed using the Shapiro-Wilk test together with visual inspection of histograms and Q-Q plots. Prespecified exploratory comparisons between regular and non-regular VTV users were limited to clinically relevant factors, including clinician experience, NICU size, practice environment, modern VTV-capable ventilator availability/use, HFOV use, use of VG or VT targeting during HFOV, and VT monitoring and documentation behavior. Categorical comparisons were performed using chi-square or Fisher's exact tests, as appropriate. Continuous comparisons were performed using t-tests or Mann-Whitney U tests according to distribution. Two-sided *P*-values < 0.05 were considered statistically significant and interpreted descriptively because of the exploratory design and the small, non-regular VTV group. Analyses were performed using Python with pandas and scipy; figures were generated using matplotlib. Because multiple exploratory comparisons were performed without formal correction for multiple testing, the risk of type I error is increased; accordingly, no adjustment (e.g., Bonferroni) was applied, and all *P*-values were interpreted descriptively, with statistically significant associations regarded as hypothesis-generating rather than confirmatory.

Ethical considerations

The survey collected clinician-reported practice data only and did not include physician-level information.

## Results

Respondent characteristics

**Figure 1:**
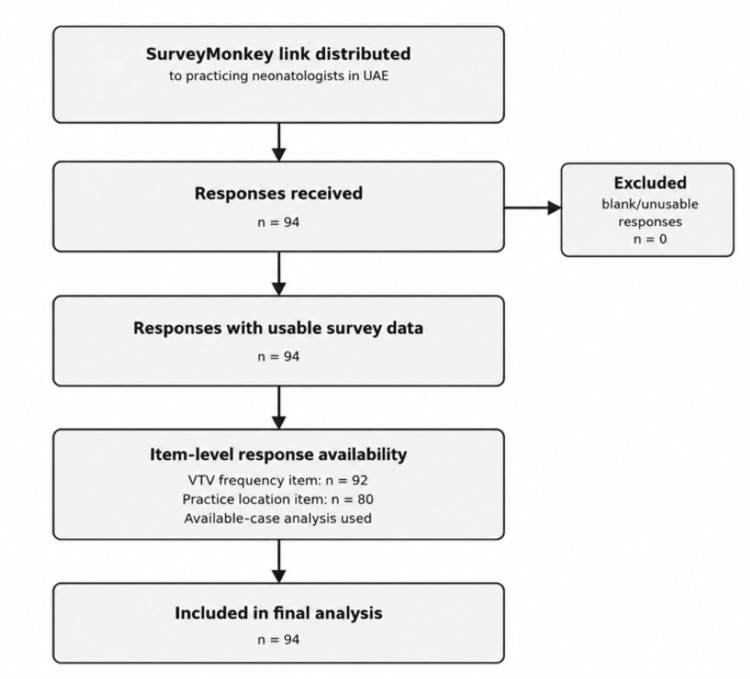
Survey response flow diagram. Note: The number of neonatologists invited was not recorded in the response file. All received responses were retained for the final analysis; item-level denominators varied across survey questions. Created by authors using Python, specifically the matplotlib plotting library (an open-source library developed and maintained by the Matplotlib Development Team/NumFOCUS; matplotlib.org). VTV, volume-targeted ventilation

A total of 94 survey responses were received and retained for the final analysis. Item-level denominators varied because not all respondents answered every survey item; therefore, available-case denominators were used throughout. Respondents represented multiple UAE practice regions, with the largest proportions from Abu Dhabi and Al Ain, and more than half reported at least 10 years of neonatology practice. Most respondents practiced in academic or training level III NICUs, and approximately half reported working in NICUs with at least 40 level II-III beds (Table 1; Figure 1).

**Table 1:** Respondent and NICU characteristics. Note: Percentages are based on non-missing responses for each domain. Northern Emirates include Ajman, Fujairah, Ras Al Khaimah, and Umm Al Quwain. VTV, volume-targeted ventilation; NICU, neonatal intensive care unit

Domain	Category	Respondents, *n* (%)
Practice location	Abu Dhabi region	33 (41.2)
	Al Ain region	18 (22.5)
	Dubai region	9 (11.2)
	Sharjah region	10 (12.5)
	Northern Emirates	10 (12.5)
Years of neonatology practice	<5	17 (21.2)
	5-10	21 (26.2)
	≥10	42 (52.5)
Practice environment	Academic/training level III NICU	54 (69.2)
	Level III without training program	21 (26.9)
	Level II NICU	3 (3.8)
Level II–III NICU beds	<20	16 (20.0)
	20-40	25 (31.2)
	≥40	39 (48.8)
VTV use group	Regular VTV user	78 (84.8)
	Non-regular VTV user	14 (15.2)

Core ventilation practices and VTV utilization

**Figure 2:**
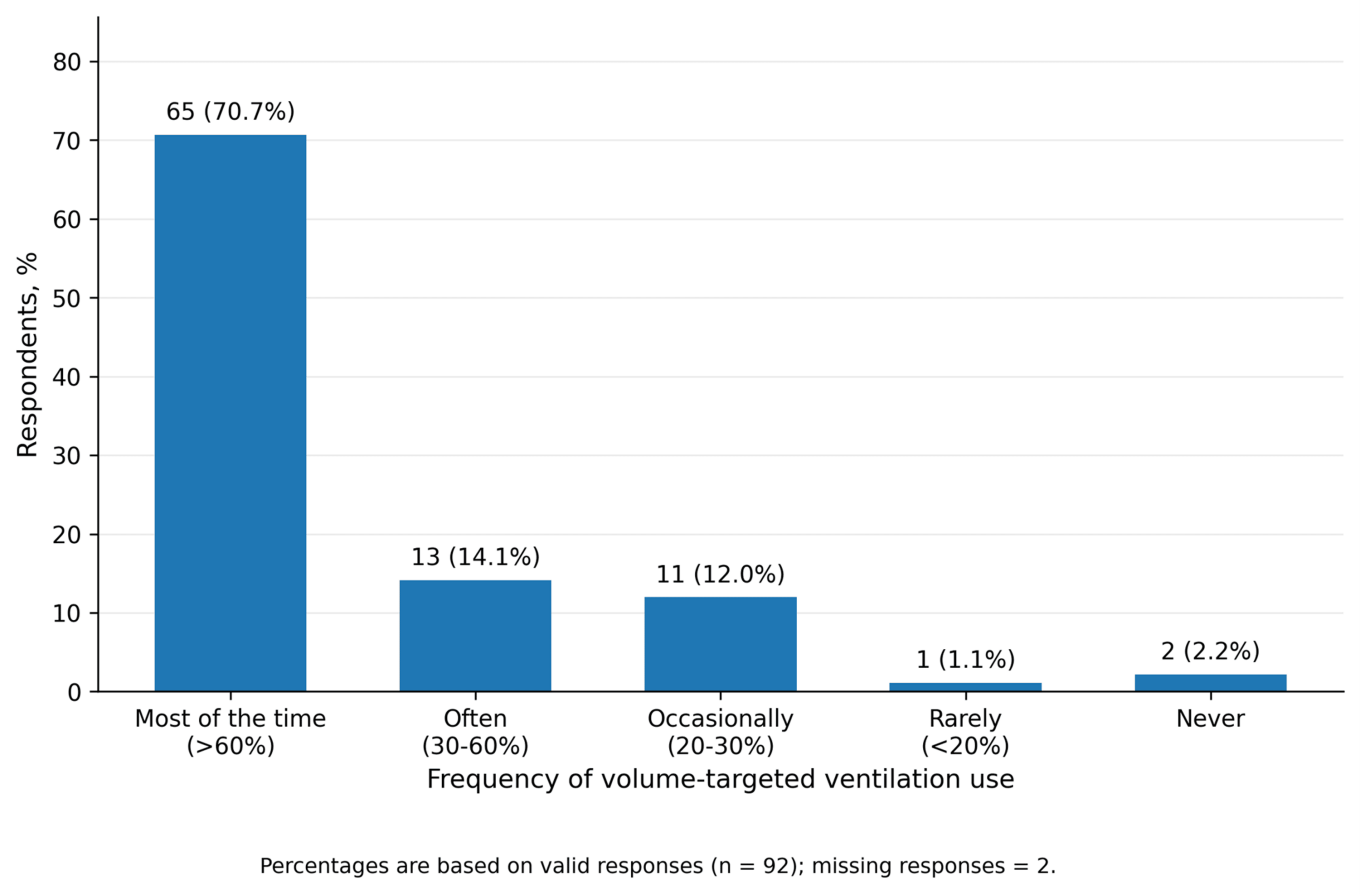
Frequency of VTV use. Note: Bars show the distribution of responses to the VTV frequency item. Percentages are calculated among respondents with available data for this item. VTV, volume-targeted ventilation

VTV was the most commonly reported invasive ventilation approach for ELBW/VLBW infants. Among respondents with available data for VTV frequency, 78 (84.8%) were classified as regular VTV users. Most respondents reported using VTV either most of the time or often, while rare or no use was uncommon (Table 2; Figure 2).

**Table 2:** Core ventilation practices and VTV utilization. Note: Percentages are based on non-missing responses for each item. Conventional ventilator items were select-all-that-apply questions and are summarized among respondents who selected at least one ventilator. The five most commonly reported ventilators are shown; less common models may be reported in the Supplementary Material. Tidal-volume targets are presented as median (IQR) because the distributions were non-normal. Severe BPD was not included because only one numerical response was available. VTV, volume-targeted ventilation; VT, tidal volume; HFOV, high-frequency oscillatory ventilation; VG, volume guarantee; RDS, respiratory distress syndrome; MAS, meconium aspiration syndrome; CDH, congenital diaphragmatic hernia

Domain	Practice/setting	Respondents, *n* (%) or summary
Most commonly reported conventional ventilators (select all that apply)	Dräger VN500	69 (75.8)
	SLE6000	34 (37.4)
	Fabian (Acutronic)	32 (35.2)
	Dräger VN600/VN800	23 (25.3)
	Maquet Servo-i	23 (25.3)
Most often used invasive mode	VTV	76 (83.5)
	Pressure-controlled/pressure-limited	10 (11.0)
	Equally often	5 (5.5)
Frequency of VTV use	Most of the time (>60%)	65 (70.7)
	Often (30%-60%)	13 (14.1)
	Occasionally (20%-30%)	11 (12.0)
	Rarely/never	3 (3.3)
Clinical timing of VTV use	Both acute and weaning phases	60 (64.5)
	Immediately after birth/acute phase only	28 (30.1)
	Weaning phase only	1 (1.1)
	Not applicable/does not use VTV	4 (4.3)
HFOV practice	Uses HFOV	78 (97.5)
VG/VT setting during HFOV	Yes	28 (36.4)
	No	49 (63.6)
VT targets	Lowest VT used during VTV (mL/kg)	4.0 (4.0-4.0); *n* = 90
	Highest VT commonly used in acute phase (mL/kg)	6.0 (6.0-6.0); *n* = 80
	Initial VT: 500 g, 24-week infant with RDS (mL/kg)	5.0 (5.0-5.0); *n* = 79
	Initial VT: term infant with MAS (mL/kg)	5.0 (4.0-5.0); *n* = 75
	Initial VT: term infant with CDH (mL/kg)	4.0 (4.0-5.0); *n* = 73

**Figure 3:**
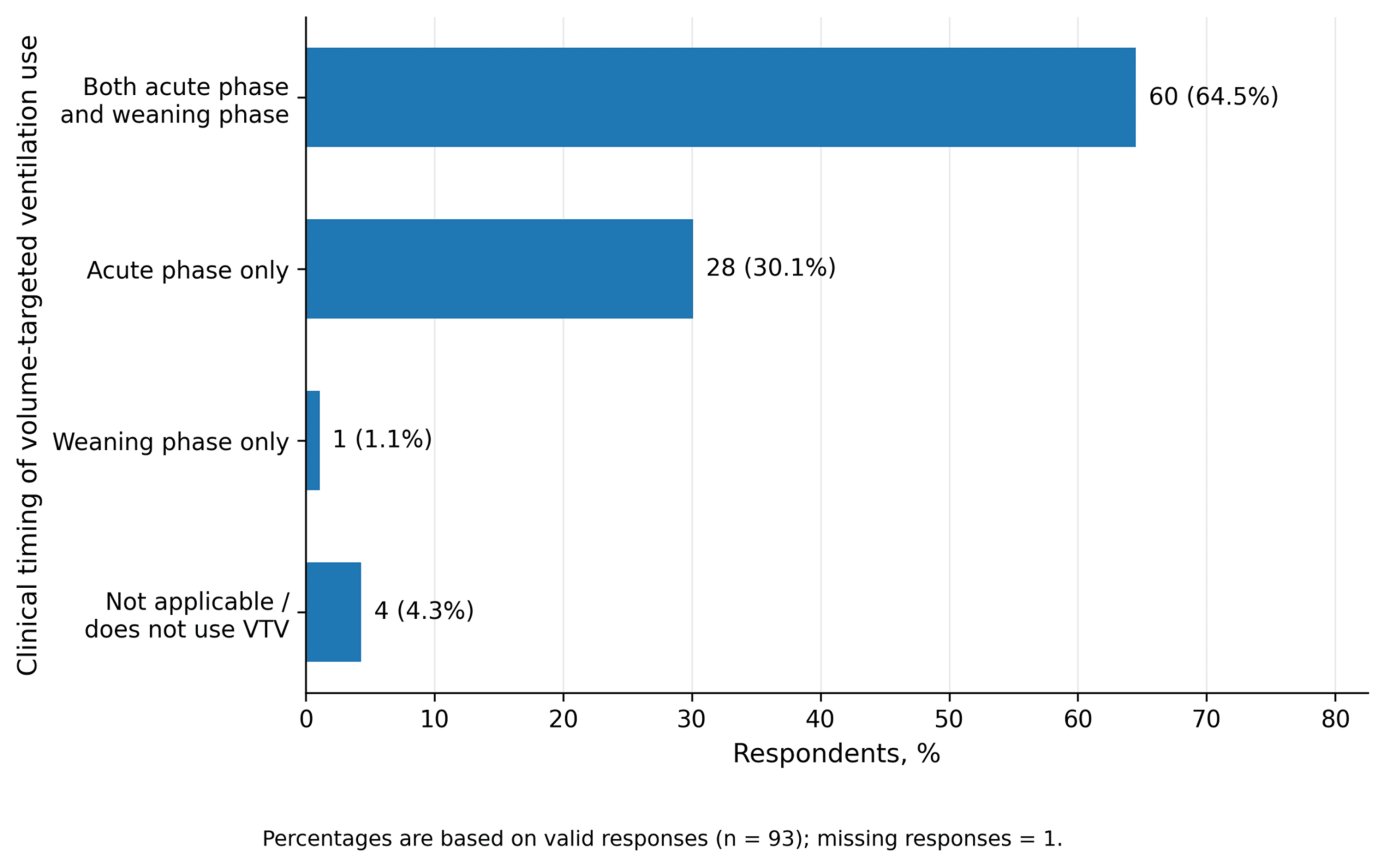
Clinical timing of VTV use. Note: Bars show whether respondents reported using VTV during the acute phase, the weaning phase, both phases, or not using VTV. VTV, volume-targeted ventilation

Clinically, VTV was most often used across both acute and weaning phases, rather than being restricted to a single phase of ventilation. HFOV was widely reported, although setting VG or a VT target during HFOV was less frequent (Table 2; Figure 3).

VT targets

**Figure 4:**
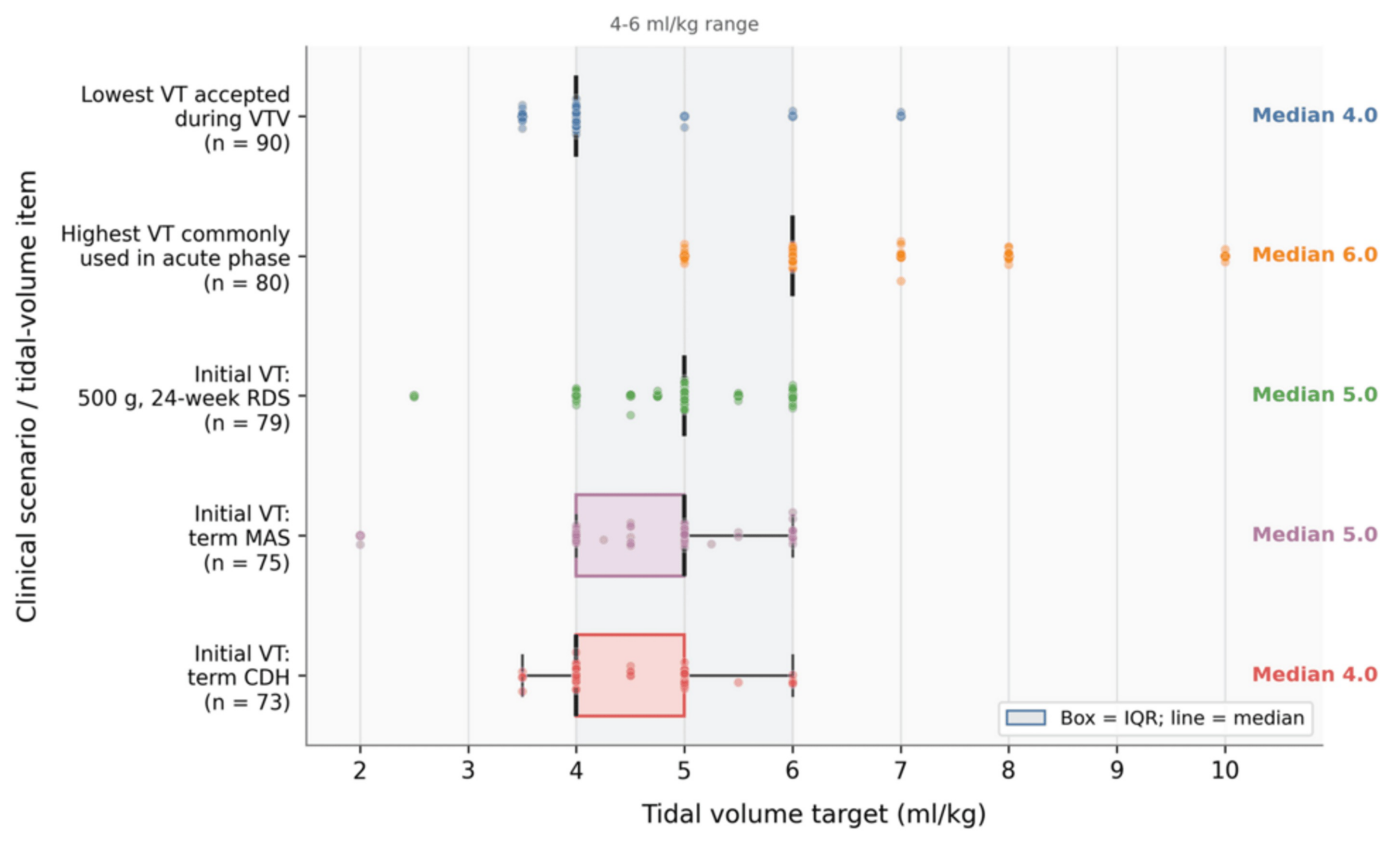
VT targets by clinical scenario. Note: Boxplots summarize the distribution of numeric tidal-volume (VT) targets. Individual points represent respondent-level values. Severe BPD was omitted because only one usable numeric response was available. VTV, volume-targeted ventilation; BPD, bronchopulmonary dysplasia; RDS, respiratory distress syndrome; MAS, meconium aspiration syndrome; CDH, congenital diaphragmatic hernia

Reported VT targets clustered within the lung-protective range typically used in neonatal ventilation. The lowest VTV target was centered around 4.0 mL/kg, the highest acute-phase target around 6.0 mL/kg, and initial targets across the selected clinical scenarios generally ranged from 4.0 to 5.0 mL/kg (Table 2; Figure 4).

Factors associated with regular VTV use

In the prespecified exploratory comparisons, regular VTV use differed by several clinician, unit, and practice-related characteristics. Regular users were more likely to have at least 10 years of neonatology practice and to work in academic or training level III NICUs. In contrast, respondents from larger NICUs were overrepresented among non-regular users in this dataset. Regular VTV use was also associated with setting volume guarantee (VG)/VT during HFOV and with always checking generated VT during pressure-controlled ventilation. Modern VTV-capable ventilator availability, HFOV use, and routine chart documentation of VT did not differ meaningfully between groups (Table 3).

**Table 3:** Prespecified factors associated with regular VTV use. Note: Regular VTV use was defined as use most of the time (>60%) or often (30%-60%), whereas non-regular use was defined as occasional, rare, or no use. Each row uses available-case denominators. Comparisons were prespecified based on their clinical relevance to VTV adoption and should be interpreted as exploratory. *P*-values are presented in APA style. VTV, volume-targeted ventilation; VT, tidal volume; NICU, neonatal intensive care unit; HFOV, high-frequency oscillatory ventilation; VG, volume guarantee

Domain	Factor	Overall, *n*/*N* (%)	Regular VTV users, *n*/*N* (%)	Non-regular VTV users, *n*/*N* (%)	P
Clinician/unit characteristics	≥10 years of neonatology practice	42/80 (52.5)	41/71 (57.7)	1/9 (11.1)	0.011
	Large NICU (≥40 level II-III beds)	39/80 (48.8)	31/71 (43.7)	8/9 (88.9)	0.013
	Academic/training level III NICU	54/78 (69.2)	51/69 (73.9)	3/9 (33.3)	0.021
Technology and advanced ventilation	Modern VTV-capable conventional ventilator available/used	85/90 (94.4)	73/76 (96.1)	12/14 (85.7)	0.171
	Uses HFOV	78/80 (97.5)	69/71 (97.2)	9/9 (100.0)	1.000
	Sets VG/VT during HFOV	28/77 (36.4)	28/69 (40.6)	0/8 (0.0)	0.045
Monitoring and documenting behavior	Always checks the generated VT during pressure-controlled ventilation	63/88 (71.6)	59/75 (78.7)	4/13 (30.8)	0.001
	Records VT in the chart always/sometimes	65/77 (84.4)	54/63 (85.7)	11/14 (78.6)	0.449

Reported barriers

**Figure 5:**
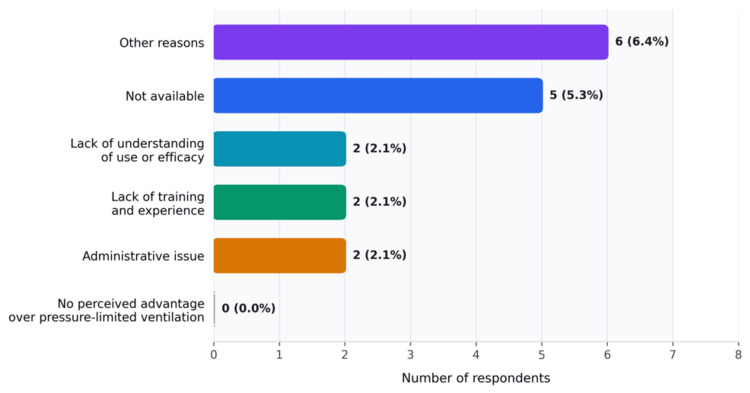
Reported barriers to VTV use. Note: Bars show the number and percentage of respondents selecting each barrier. The response option “No, I use it” was excluded because it does not represent a barrier. VTV, volume-targeted ventilation

Reported barriers to VTV use were relatively infrequent. The most common barriers were other respondent-specified reasons and equipment unavailability, whereas lack of training, lack of understanding, and administrative issues were selected less often. These findings are summarized in Figure 5.

Detailed item-level distributions and extended analyses are provided in Tables 4-8.

**Table 4:** Respondent and NICU characteristics according to regular volume-targeted ventilation use. Note: Values are *n* (%). Percentages are calculated using non-missing responses for each variable. VTV, volume-targeted ventilation; NICU, neonatal intensive care unit; HFOV, high-frequency oscillatory ventilation

Variable/domain	Category/item	Overall	Regular VTV users	Non-regular VTV users	P
Years of neonatology practice	<5 years	19 (23.8)	15 (21.1)	4 (44.4)	0.098
	5-10 years	19 (23.8)	15 (21.1)	4 (44.4)	
	10-15 years	9 (11.2)	8 (11.3)	1 (11.1)	
	15-20 years	16 (20.0)	16 (22.5)	0 (0.0)	
	>20 years	17 (21.2)	17 (23.9)	0 (0.0)	
Level II-III NICU beds	<10	7 (8.8)	7 (9.9)	0 (0.0)	0.041
	10-20	9 (11.2)	8 (11.3)	1 (11.1)	
	20-40	25 (31.2)	25 (35.2)	0 (0.0)	
	40-60	27 (33.8)	20 (28.2)	7 (77.8)	
	>60	12 (15.0)	11 (15.5)	1 (11.1)	
Primary practice location	Abu Dhabi region	33 (41.2)	26 (36.6)	7 (77.8)	0.240
	Al Ain region	18 (22.5)	18 (25.4)	0 (0.0)	
	Dubai region	9 (11.2)	7 (9.9)	2 (22.2)	
	Sharjah region	10 (12.5)	10 (14.1)	0 (0.0)	
	Ajman region	4 (5.0)	4 (5.6)	0 (0.0)	
	Fujairah region	4 (5.0)	4 (5.6)	0 (0.0)	
	Ras Al-Khaimah region	1 (1.2)	1 (1.4)	0 (0.0)	
	Umm Al Quwain region	1 (1.2)	1 (1.4)	0 (0.0)	
Practice environment	Level III, no residents or fellows (i.e., no training program)	21 (26.9)	15 (21.7)	6 (66.7)	0.037
	Academic level III or higher NICU with residency and a fellowship program	47 (60.3)	44 (63.8)	3 (33.3)	
	Academic level III or higher NICU with residency (no fellowship programs)	7 (9.0)	7 (10.1)	0 (0.0)	
	Level II NICU	3 (3.8)	3 (4.3)	0 (0.0)	
Uses high-frequency mechanical ventilation	Yes	78 (97.5)	69 (97.2)	9 (100.0)	1.000
	No	2 (2.5)	2 (2.8)	0 (0.0)

**Table 5:** Ventilator availability and ventilation practices according to regular volume-targeted ventilation use. Note. Values are *n* (%). Multi-select items are summarized among respondents with at least one response to the corresponding question. Ventilator items allowed multiple responses; therefore, percentages may exceed 100% when summed across items. VTV, volume-targeted ventilation; HFOV, high-frequency oscillatory ventilation; VG, volume guarantee; VT, tidal volume; PRVC, pressure-regulated volume control

Variable/domain	Category/item	Overall	Regular VTV users	Non-regular VTV users	P
Conventional ventilator used	Dräger Babylog 8000/8000 Plus	18 (20.0)	16 (21.1)	2 (14.3)	0.727
	Dräger VN500	68 (75.6)	56 (73.7)	12 (85.7)	0.503
	Dräger VN600/VN800	23 (25.6)	22 (28.9)	1 (7.1)	0.105
	Fabian (Acutronic)	31 (34.4)	22 (28.9)	9 (64.3)	0.015
	Maquet Servo-i	23 (25.6)	15 (19.7)	8 (57.1)	0.006
	CareFusion Avea	0 (0.0)	0 (0.0)	0 (0.0)	-
	GE Carestation	0 (0.0)	0 (0.0)	0 (0.0)	-
	Puritan Bennett	1 (1.1)	0 (0.0)	1 (7.1)	0.156
	Maquet Servo-n (neonatal)	10 (11.1)	10 (13.2)	0 (0.0)	0.351
	SLE6000	34 (37.8)	26 (34.2)	8 (57.1)	0.104
	Other conventional ventilators	3 (3.3)	3 (3.9)	0 (0.0)	1.000
Most often used invasive mode	Volume targeted Ventilation (e.g., Volume Guarantee, PRVC, volume control)	76 (83.5)	68 (87.2)	8 (61.5)	0.002
	Pressure-controlled/pressure-limited	10 (11.0)	5 (6.4)	5 (38.5)	
	Equally often	5 (5.5)	5 (6.4)	0 (0.0)	
Timing of VTV use	Immediately after birth (acute phase)	27 (29.3)	26 (33.3)	1 (7.1)	<0.001
	Weaning phase	1 (1.1)	0 (0.0)	1 (7.1)	
	Both	60 (65.2)	51 (65.4)	9 (64.3)	
	N/A (If you do not use volume-targeted conventional ventilation)	4 (4.3)	1 (1.3)	3 (21.4)	
HFOV machine used	HFOV machine: Dräger	69 (87.3)	61 (85.9)	8 (100.0)	0.586
	HFOV machine: SensorMedics	26 (32.9)	20 (28.2)	6 (75.0)	0.014
	HFOV machine: Fabian	11 (13.9)	11 (15.5)	0 (0.0)	0.591
	HFOV machine: other	11 (13.9)	11 (15.5)	0 (0.0)	0.591
	HFOV machine: not applicable	2 (2.5)	2 (2.8)	0 (0.0)	1.000
Sets VG/VT with HFOV	Yes	28 (35.0)	28 (39.4)	0 (0.0)	0.043
	No	49 (61.2)	41 (57.7)	8 (88.9)	
	N/A	3 (3.8)	2 (2.8)	1 (11.1)

**Table 6:** Clinical timing of VTV use and tidal-volume targets according to regular VTV use. Note: Categorical values are presented as *n* (%). Tidal-volume variables are presented as mean (SD) or median (IQR), as appropriate based on Shapiro-Wilk normality testing, with all numerical summaries rounded to one decimal place. Clinical timing items may be multi-select. Tidal-volume values are expressed in mL/kg. VTV, volume-targeted ventilation; VT, tidal volume; MAS, meconium aspiration syndrome; RDS, respiratory distress syndrome; IQR, interquartile range

Variable/domain	Category/item	Overall	Regular VTV users	Non-regular VTV users	P
Clinical timing of VTV use	Immediately after birth (acute phase)	27 (29.3)	26 (33.3)	1 (7.1)	<0.001
	Weaning phase	1 (1.1)	0 (0.0)	1 (7.1)	
	Both	60 (65.2)	51 (65.4)	9 (64.3)	
	N/A (If you do not use volume-targeted conventional ventilation)	4 (4.3)	1 (1.3)	3 (21.4)	
Tidal volume targets	Lowest VT used during VTV, mL/kg; median (IQR)	4.0 (4.0-4.0)	4.0 (4.0-4.0)	4.0 (4.0-4.0)	0.021
	Highest VT commonly used in the acute phase, mL/kg; median (IQR)	6.0 (6.0-6.0)	6.0 (6.0-6.0)	6.0 (6.0-6.0)	0.117
	Initial VT: one-day-old 500 g, 24-week infant with RDS, mL/kg; median (IQR)	5.0 (5.0-5.0)	5.0 (5.0-5.0)	6.0 (5.2-6.0)	0.011
	Initial VT: one-day-old 3500 g term infant with MAS/pneumonia, mL/kg; median (IQR)	5.0 (4.0-5.0)	5.0 (4.0-5.0)	5.0 (5.0-5.0)	0.219
	Initial VT: one-day-old 2800 g term infant with RDS/pneumonia, mL/kg; median (IQR)	4.0 (4.0-5.0)	4.0 (4.0-5.0)	4.0 (4.0-4.0)	0.085

**Table 7:** Tidal-volume monitoring, documentation, perceived benefits, and barriers according to regular VTV use. Note: Values are *n* (%). Multi-select perceived benefits and barriers are summarized among respondents with at least one response to the corresponding item block. Comparisons are exploratory because multiple survey items were tested. Benefit and barrier items may be multi-select; therefore, percentages may exceed 100% across items. VTV, volume-targeted ventilation; VT, tidal volume; PCV, pressure-controlled ventilation; BPD, bronchopulmonary dysplasia; IVH, intraventricular hemorrhage; PVL, periventricular leukomalacia

Variable/domain	Category/item	Overall	Regular VTV users	Non-regular VTV users	P
Checks generated VT during pressure-controlled ventilation	Always	63 (68.5)	59 (75.6)	4 (28.6)	0.003
	Sometimes	20 (21.7)	12 (15.4)	8 (57.1)	
	Occasionally	5 (5.4)	4 (5.1)	1 (7.1)	
	None	4 (4.3)	3 (3.8)	1 (7.1)	
Records VT in the medical record/flowchart	Always	34 (37.4)	31 (40.3)	3 (21.4)	0.069
	Sometimes	31 (34.1)	23 (29.9)	8 (57.1)	
	Occasionally	12 (13.2)	9 (11.7)	3 (21.4)	
	None	14 (15.4)	14 (18.2)	0 (0.0)	
Perceived benefits of VTV	Improved survival for VLBW infants	52 (65.0)	45 (63.4)	7 (77.8)	0.483
	Lower ventilation-induced lung injury/lower BPD	73 (91.2)	64 (90.1)	9 (100.0)	1.000
	Lower severe IVH and PVL	38 (47.5)	37 (52.1)	1 (11.1)	0.031
	Shorter duration of mechanical ventilation	45 (56.2)	38 (53.5)	7 (77.8)	0.286
	Lower rate of severe disability	24 (30.0)	23 (32.4)	1 (11.1)	0.266
	Other perceived benefits	5 (6.2)	4 (5.6)	1 (11.1)	0.458
Barriers to VTV use	No perceived advantage	0 (0.0)	0 (0.0)	0 (0.0)	-
	Not available	5 (6.6)	5 (7.4)	0 (0.0)	1.000
	Lack of understanding of use/efficacy	2 (2.6)	2 (2.9)	0 (0.0)	1.000
	Lack of training and experience	2 (2.6)	2 (2.9)	0 (0.0)	1.000
	Administrative issue	2 (2.6)	2 (2.9)	0 (0.0)	1.000
	No barrier: I use it	65 (85.5)	57 (83.8)	8 (100.0)	0.594
	Other barrier	6 (7.9)	5 (7.4)	1 (12.5)	0.499

**Table 8:** Exploratory logistic regression for factors associated with regular VTV use. Outcome was regular VTV use. The adjusted model included >10 years of practice, a large NICU, an academic/training NICU, and always checking generated VT during pressure-controlled ventilation. Complete-case regression sample: *n* = 77. Regression results should be interpreted as exploratory because of the modest sample size and limited number of non-regular VTV users. OR, odds ratio; CI, confidence interval; VTV, volume-targeted ventilation; VT, tidal volume; NICU, neonatal intensive care unit

Predictor	Unadjusted OR (95% CI)	P	Adjusted OR (95% CI)	P
>10 years of practice	10.8 (1.3-90.9)	.029	26.9 (0.8-883.5)	0.064
Large NICU (≥40 level II-III beds)	0.1 (0.0-0.8)	.033	0.0 (0.0-0.3)	0.010
Academic/training NICU	5.6 (1.3-24.6)	.024	23.2 (1.6-342.1)	0.022
Always checks the generated VT during PCV	12.4 (2.3-65.9)	.003	65.0 (3.1-1352.6)	0.007
Modern VTV-capable conventional ventilator	2.7 (0.3-29.2)	.412	-	-

## Discussion

The widespread utilization of VTV as a primary mode of invasive respiratory support for ELBW and VLBW infants is a major finding of this study, with 84.8% of surveyed neonatologists in the UAE classified as regular VTV users. This high adoption rate is striking when compared to international data. Gupta and Keszler demonstrated a stark geographic contrast in North America, with 81% of Canadian neonatologists utilizing VTV as a primary mode compared to only 39% in the United States [1]. The UAE’s adoption rate of 78 (84.8%) closely mirrors progressive Canadian practices and noticeably surpasses the 67.2% routine VTV utilization rate recently reported in neighboring Saudi Arabia [9]. Furthermore, the barriers to implementation highlight key geographic differences. While a lack of understanding regarding VTV was identified as the primary barrier to adoption in the United States (49%) [1], UAE respondents primarily reported equipment unavailability as their most significant hurdle. This finding perfectly aligns with the Saudi Arabian cohort, where the limited availability of appropriate ventilator devices was also cited as the leading obstacle (49%) [9]. This regional similarity suggests a high level of clinical acceptance in the Middle East that is occasionally constrained by institutional resource limitations rather than practitioner reluctance.

A core component of a successful VTV application is the individualization of VT targets. As emphasized by expert guidelines, *one size does not fit all* when it comes to neonatal ventilation, and VT must be tailored to the infant's size, postnatal age, and underlying lung pathophysiology [8]. In our study, the reported median lowest and highest VT targets (4.0 and 6.0 mL/kg, respectively) align well with general lung-protective strategies. However, the scenario-based responses reveal some discordance with specific evidence-based recommendations, showing a tendency to default to standard volumes regardless of pathology. This difficulty in translating physiological evidence into bedside practice reflects a global phenomenon; for instance, the failure to adequately increase VT for MAS was observed in our cohort and directly mirrors findings from both North America and Saudi Arabia, where only 17% and 13% of respondents, respectively, selected the appropriately higher evidence-based VT targets for MAS [1,9]. Similarly, lung mechanics evolve significantly over time; the VT required to maintain normocapnia in extremely small infants rises progressively during the first three weeks of life due to acquired tracheomegaly and increased alveolar dead space [10]. Practitioners must continuously reassess and titrate VT upwards as chronic lung disease evolves, yet Al Qurashi et al. found only a 29% agreement with appropriate VT selection for evolving BPD [9]. This reinforces that the failure to adjust VT for specific clinical scenarios remains a universal hurdle in optimizing VTV efficacy.

For term infants, the median selected VT of 5.0 mL/kg for MAS and 4.0 mL/kg for CDH reflects an understanding of varying lung mechanics, albeit with room for optimization. Infants with MAS typically require a higher VT (5.5-6.0 mL/kg) due to increased alveolar dead space and air trapping, while infants with CDH require a lower VT (4.0-4.5 mL/kg) to avoid volutrauma in hypoplastic lungs while maintaining necessary alveolar minute ventilation [8]. The tendency of some neonatologists to default to a standard 4.0 to 5.0 mL/kg target underscores a global phenomenon reported by Gupta and Keszler, where the failure to adjust VT for specific clinical scenarios remains a significant hurdle in optimizing VTV efficacy [1].

The clinical rationale for targeting specific volumes is heavily rooted in achieving carbon dioxide (CO₂) stability and mitigating the risks of hypocarbia. VTV dynamically adjusts peak inspiratory pressure (PIP) in response to breath-to-breath changes in lung compliance, thereby preventing inadvertent hyperventilation and volutrauma [11]. A randomized controlled trial by Cheema et al. demonstrated that VG ventilation significantly reduced the incidence of hypocarbia compared to synchronous intermittent positive pressure ventilation (SIPPV) alone (32% vs. 57%), particularly during the critical initial stabilization period [11]. By maintaining a stable VT, clinicians can stabilize cerebral blood flow and reduce the risk of severe neurologic injuries such as intraventricular hemorrhage and periventricular leukomalacia [12].

An intriguing finding from our survey is the discrepancy between the high utilization of HFOV (78 (97.5%)) and the relatively low adoption of VG/VT targeting during HFOV (28 (36.4%)). While HFOV alone is a well-established lung-protective strategy that utilizes VTs smaller than the anatomical dead space to minimize barotrauma, the addition of VG to HFOV (HFOV-VG) represents a promising technological advancement [12]. HFOV-VG allows clinicians to set a target high-frequency VT (VThf), automatically adjusting the amplitude to maintain consistent volume delivery despite rapidly changing respiratory mechanics [13]. This tight control reduces VThf fluctuations, prevents out-of-target pCO₂ values, and minimizes hypoxic attacks [13]. The lower adoption of HFOV-VG in our cohort likely reflects the novelty of the modality, a lack of established consensus on optimal VThf targets, and a need for further training, as setting appropriate VThf requires close monitoring of DCO₂ levels and pCO₂ trends [13].

Limitations

Several limitations should be considered when interpreting the findings of this study. First, because the exact number of invited neonatologists was not recorded, the overall response rate remains unknown, limiting the ability to assess potential non-response bias. Although a precise denominator is unavailable, the neonatology workforce in the UAE is relatively small and can be approximated at roughly 120-150 practicing clinicians on the basis of the number of level II-III NICUs nationally; against this estimate, the 94 analyzed responses likely represent a substantial proportion of the national workforce. Nonetheless, the possibility of non-response bias-whereby clinicians more engaged with VTV may have been more inclined to respond-cannot be excluded and could have influenced the reported adoption rates. Second, as is inherent in survey research, the data rely on self-reported practices rather than direct observation of bedside care, which may not always reflect actual clinical application [1]. Third, there is a risk of selection bias; neonatologists with a specific interest, training, or expertise in advanced lung-protective strategies may have been more likely to participate, which could potentially inflate the reported VTV adoption rates. Consequently, the adoption rates reported here should be interpreted as estimates that may not be fully representative of all neonatologists practicing in the UAE. Finally, the unexpectedly high prevalence of VTV use resulted in a very small subgroup of non-regular VTV users (*n* = 14). This small sample size limits the statistical power to conduct robust comparative analyses or to fully characterize the specific practitioner factors associated with non-adoption. In addition, numerous hypothesis tests were performed across the exploratory analyses; some statistically significant findings may therefore reflect chance (type I error), and all such associations should be regarded as hypothesis-generating and confirmed in larger, adequately powered studies.

## Conclusions

This study provides the first comprehensive characterization of VTV practices among neonatologists in the UAE, revealing a robust adoption rate. With 78 (84.8%) of respondents utilizing VTV as their primary mode of invasive respiratory support, the UAE aligns with the most progressive international benchmarks for neonatal care. Furthermore, the primary barrier to implementation in the region is equipment unavailability rather than a lack of clinical understanding. This demonstrates a mature clinical acceptance of advanced lung-protective strategies that is currently constrained primarily by institutional resource limitations rather than practitioner reluctance.

Despite the high prevalence of VTV use, our scenario-based findings highlight a critical need for ongoing quality improvement regarding the individualization of VT settings. While practitioners correctly identified safe general boundaries, there remains a notable tendency to apply a standardized VT target across highly varied clinical conditions, thereby overlooking the distinct pathophysiologies of extremely small infants, MAS, and evolving chronic lung disease. Moving forward, regional initiatives should focus on addressing equipment disparities, expanding the integration of VG during HFOV, and providing targeted education to ensure ventilation targets are precisely tailored to each infant's unique and evolving lung mechanics.

## Appendices

Appendix: Survey questionnaire

**Table 9:** Complete survey instrument administered to practicing neonatologists in the United Arab Emirates, presenting all items and their response options. ELBW, extremely low birth weight; VLBW, very low birth weight; PRVC, pressure-regulated volume control; RDS, respiratory distress syndrome; MAS, meconium aspiration syndrome; CDH, congenital diaphragmatic hernia; SIMV, synchronized intermittent mandatory ventilation; PS, pressure support; PSV, pressure support ventilation; BPD, bronchopulmonary dysplasia; IVH, intraventricular hemorrhage; PVL, periventricular leukomalacia; NICU, neonatal intensive care unit; HFOV, high-frequency oscillatory ventilation; VG, volume guarantee

Item	Survey question	Response options
Q1	Which conventional ventilator(s) do you use in ELBW / VLBW infants? (Check all that apply.)	• Draeger Babylog 8000 (or 8000 Plus) • Draeger VN500 • Draeger VN600 / VN800 • Fabian (Acutronic) • Maquet Servo-i • Carefusion (Avea) • GE Carestation • Puritan Bennett • Maquet Servo-n (Neonatal) • SLE6000 • Other (please specify)
Q2	Which mode of ventilation do you use most often in ELBW / VLBW infants (if invasive ventilation is required)?	• Pressure-controlled / pressure-limited • Volume-targeted ventilation (e.g., Volume Guarantee, PRVC, Volume Control) • Equally often
Q3	How often do you use volume-targeted ventilation?	• Never • Rarely (<20%) • Occasionally (20–30%) • Often (30–60%) • Most of the time (>60%)
Q4	If you use volume-targeted ventilation in your center, when is it used?	• Immediately after birth (acute phase) • Weaning phase • Both • N/A (if you don't use volume-targeted conventional ventilation)
Q5	When you use pressure-controlled ventilation, do you / your center look at the generated tidal volume?	• Always • Occasionally • Sometimes • None
Q6	When using pressure-controlled ventilation, do you / your center record tidal volume in your medical record / flow chart (electronic or paper records)?	• Always • Occasionally • Sometimes • None
Q7	If you decided to use / shift to pressure-controlled mechanical ventilation, what makes you use it?	• High leak >70% • Constant pressure above target limit • Both A and B • I use it first as initial mode • None of the above
Q8	When using volume-targeted ventilation, what is the lowest tidal volume (cannot go below it) that you can use?	• 3.5 mL/kg • 4 mL/kg • 5 mL/kg • 6 mL/kg • 7 mL/kg • Do not use volume-targeted ventilation
Q9	When using volume-targeted ventilation in the acute phase, what is the largest tidal volume you most commonly use? (Applies to any patient with any diagnosis.)	• 5 mL/kg or less • 6 mL/kg • 7 mL/kg • 8 mL/kg • 9 mL/kg • 10 mL/kg • Do not use volume-targeted ventilation
Q10	When using volume-targeted modes, what tidal volume would you choose initially for the following infants in mL/kg? (Answer each field.)	• One-day-old 500 g, 24-week baby with RDS • One-day-old 3500 g term baby with MAS (meconium aspiration syndrome) • One-day-old 2800 g term infant with CDH (congenital diaphragmatic hernia)
Q11	Which modes of synchronized conventional ventilation do you use most often during the acute phase in preterm infants with RDS? Please rank them in order of frequency (1 = most frequent, 2 = second, etc.). (Answer each field.)	• Assist/control (AC) • Synchronized intermittent mandatory ventilation (SIMV) • SIMV with pressure support (SIMV + PS) • Pressure support ventilation (PSV) • Other
Q12	Which mode of ventilation do you use during the weaning phase? Please rank in order of frequency (1 = most frequent, 2 = secondary, etc.). (Answer each field.)	• Assist/control (A/C) • SIMV • SIMV with pressure support (SIMV + PS) • Pressure support ventilation (PSV) • Other (specify)
Q13	If you use volume-targeted ventilation, please specify the main reasons (advantages) to do so. (List as many benefits as you think of.)	• Improved survival for VLBW infants using volume-targeted vs. pressure-limited ventilation • Lower ventilation-induced lung injury (lower BPD) • Lower severe IVH and PVL • Shorter duration of mechanical ventilation • Lower rate of severe disability • Other (please specify)
Q14	If you do not use volume-targeted ventilation, please specify the reasons for not using it. (List as many as applicable.)	• No advantage of volume-targeted over pressure-limited ventilation • Not available • Lack of understanding of how to use it or its efficacy • Lack of training and experience • Administrative issue • No, I use it • Other (please specify)
Q15	Total number of level II–III beds in your NICU.	• <10 • 10–20 • 20–40 • 40–60 • >60
Q16	Primary practice location in the United Arab Emirates.	• Abu Dhabi region • Al Ain region • Dubai region • Sharjah region • Ajman region • Ras Al-Khaimah region • Fujairah region • Al Thaid region • Umm Al Quwain region
Q17	How long have you practiced neonatology? (Excluding training experience.)	• <5 years • 5–10 years • 10–15 years • 15–20 years • >20 years
Q18	What is your primary practice environment?	• Level 2 NICU • Level 3, no residents or fellows (i.e., no training program) • Academic level 3 or higher NICU with residency (no fellowship program) • Academic level 3 or higher NICU with residency and a fellowship program
Q19	Do you use high-frequency mechanical ventilation in your unit? (If yes, go to next question.)	• Yes • No
Q20	If yes, which machine do you use? (Multiple select.)	• Draeger • SensorMedics • Fabian • Other • N/A
Q21	If you do use HFOV, do you set a VG (Volume Guarantee) with it?	• Yes • No • N/A
